# Overexpression of VEGF_121_, but not VEGF_165_ or FGF-1, improves oxygenation in MCF-7 breast tumours

**DOI:** 10.1038/sj.bjc.6601539

**Published:** 2004-01-20

**Authors:** B M Fenton, S F Paoni, W Liu, S-Y Cheng, B Hu, I Ding

**Affiliations:** 1 Department of Radiation Oncology, University of Rochester Medical Center, Box 704, Rochester, NY 14642, USA; 2 Department of Pathology, University of Pittsburgh Cancer Institute, Pittsburgh, PA 15213, USA

**Keywords:** antiangiogenic, hypoxia, image analysis, perfusion, tumour vasculature

## Abstract

Vascular endothelial growth factor (VEGF) is an intensively studied molecule that has significant potential, both in stimulating angiogenesis and as a target for antiangiogenic approaches. We utilised MCF-7 breast cancer cells transfected with either of two of the major VEGF isoforms, VEGF_121_ or VEGF_165_, or fibroblast growth factor-1 (FGF-1) to distinguish the effects of these factors on tumour growth, vascular function, and oxygen delivery. While each transfectant demonstrated substantially increased tumorigenicity and growth rate compared to vector controls, only VEGF_121_ produced a combination of significantly reduced total and perfused vessel spacing, as well as a corresponding reduction in overall tumour hypoxia. Such pathophysiological effects are of potential importance, since antiangiogenic agents designed to block VEGF isoforms could in turn result in the development of therapeutically unfavourable environments. If antiangiogenic agents are also combined with conventional therapies such as irradiation or chemotherapy, microregional deficiencies in oxygenation could play a key role in ultimate therapeutic success.

Vascular endothelial growth factor (VEGF), perhaps the most critical regulator of angiogenesis in both tumours and normal tissue, is regulated by numerous factors, most notably tissue oxygen level (Shweiki *et al*, 1992). Vascular endothelial growth factor exists as six alternatively spliced isoforms, predominantly VEGF_121_ and VEGF_165_ (Ferrara, 1999), and a number of previous workers have studied vascular changes in tumour lines transfected to overexpress specific isoforms. An almost universal finding among all of the different isoforms has been that overexpression enhances tumorigenesis and tumour progression (Bicknell, 1997). In MCF-7 human breast carcinomas specifically, which normally produce low levels of VEGF, VEGF_121_ transfectants formed faster growing, more vascularised tumours in comparison to wild type (Zhang *et al*, 1995). *In vitro* and *in vivo*, VEGF_121_-transfected MCF-7 tumour cells were shown to be much more tumorigenic and angiogenic than VEGF_165_, perhaps due to the enhanced ability of the VEGF_121_ isoform to freely diffuse from the cells producing it (Zhang *et al*, 2000). Our previous studies have also shown that overexpression of VEGF_121_ or VEGF_165_ by oestrogen-dependent MCF-7 breast cells stimulates breast tumour formation and neovascularisation in an oestrogen-independent fashion in ovariectomised mice, in the absence of 17 *β*-estradiol treatment (Guo *et al*, 2003). These findings suggested that upregulation of VEGF in oestrogen-dependent breast cancer contributes to the acquisition of oestrogen-independent cancer growth by stimulating tumour angiogenesis and progression through both autocrine and paracrine mechanisms.

Findings in other tumour cell lines have been somewhat mixed. Using transformed murine fibrosarcoma cells (that initially lack VEGF) to specifically express each of the isoforms, it was found that only VEGF_164_ (the murine version of VEGF_165_) could fully rescue tumour growth (Grunstein *et al*, 2000). In this study, vascular densities were unchanged in either the VEGF_120_ or VEGF_165_ transfectants compared to vector controls. In the WM1341B melanoma cell line, however, VEGF_165_ produced much more richly vascularised tumours in transfectants, despite the fact that VEGF_121_ was the predominant isoform in parental cell lines (Yu *et al*, 2002). In gliomas, different VEGF isoforms demonstrated different biological activities than each other at the same site, as well as different activities for the same isoform when implanted at different sites (Guo *et al*, 2001).

Although the effects of VEGF_121_ and VEGF_165_ have been studied in a range of tumour models, techniques have not been available for quantifying the corresponding alterations in tumour blood flow and oxygenation until fairly recently. Since several promising antiangiogenic strategies rely on blocking either VEGF or its receptors (Gerber *et al*, 2000; Bruns *et al*, 2002), such accompanying pathophysiological changes are clearly of interest. Reductions or enhancements in tumour oxygenation could be especially important when combining antiangiogenic agents with conventional therapies, such as radiotherapy and chemotherapy, each of which directly depends on microregional tumour blood flow and oxygenation. The current work utilised MCF-7 breast cancer cells transfected with either VEGF_121_ or VEGF_165_. In addition, since FGF-1 has been similarly associated with highly vascularised tumours (Zhang *et al*, 1997), FGF-1-overexpressing transfectants were included for comparison. Using a combination of immunohistochemistry and image analysis techniques, four pathophysiological indices were determined: (1) total vessel spacing, (2) perfused vessel spacing, (3) % vascular area, and (4) overall tumour hypoxia. Results demonstrate that among these angiogenic growth factors, only VEGF_121_ overexpression produced significant alterations in overall tumour oxygenation.

## MATERIALS AND METHODS

### Cell lines and reagents

MCF-7 cells were obtained from American Tissue Culture Collection (ATCC, Rockville, MD, USA), and MCF-7 cells that stably express VEGF or FGF-1 were generated by transfecting MCF-7 cells with VEGF_121_, VEGF_165_, or FGF-1 cDNA. The clones that highly expressed exogenous VEGF_121_, VEGF_165_, or FGF-1 were expanded and characterised by methods described previously (Zhang *et al*, 1997; Guo *et al*, 2001; Guo *et al*, 2003). We detected similar levels of VEGF protein in the total cell lysates of both VEGF_121_- and VEGF_165_-expressing cells by Western blotting using an anti-VEGF antibody. In a 48 h cell culture, VEGF_121_-expressing cells secreted 296.6 ng VEGF ml^−1^ 10^6^ cells^−1^ and VEGF_165_-expressing cells produced 382.3 ng VEGF ml^−1^ 10^6^ cells^−1^ into the conditioned media, as determined by VEGF ELISA assays. We also obtained three additional VEGF-expressing cell clones in each class that express VEGF at similar levels (Guo *et al*, 2003). Human MCF-7 breast tumours were grown in the mammary fat pads of ovariectomised female nude mice as described previously (Guo *et al*, 2001). Briefly, 1 × 10^7^ cells were inoculated into the mammary fat pads of 7–8-week old, ovariectomised female nude mice that were implanted with 17-*β* estradiol 60-day slow release pellets (Innovative Research of America, Sarasota, FL, USA). The volumes of the tumours were measured using calipers and the formula ½*ab*^2^ (where *a* and *b* are the major and minor tumour dimensions).

### DiOC_7_ perfusion marker and EF5 hypoxic marker

To visualise blood vessels open to flow, an intravascular stain, DiOC_7_, was injected 1 min prior to freezing to preferentially stain cells adjacent to the vessels (Fenton *et al*, 1999). Localised areas of tumour hypoxia were assessed in frozen tissue sections by immunohistochemical identification of sites of 2-nitroimidazole metabolism (EF5 binding) (Fenton *et al*, 1999). EF5 (from NCI) was injected i.v. 1 h before tumour freezing, at which time the EF5 is well distributed throughout even poorly perfused regions of the tumour (Fenton *et al*, 2001). Regions of high EF5 metabolism were visualised immunohistochemically using a Cy3 fluorochrome conjugated to the ELK3-51 antibody, which is extremely specific for the EF5 drug adducts that form when the drug is incorporated by hypoxic cells (Lord *et al*, 1993).

### Immunohistochemistry and image analysis

Tumour sections were imaged using a × 20 objective, digitised (Sony DXC9000 3CCD camera), background-corrected, and image-analysed using Image-Pro software (Media Cybernetics, Silver Spring, MD, USA) (Fenton *et al*, 1999). Colour image montages from 16 adjacent microscope fields in each of four tumour regions (encompassing roughly 15 mm^2^) were automatically acquired and digitally combined under three different staining conditions. First, images of the DiOC_7_ were obtained immediately after the frozen sections were sliced on the cryostat. Following staining, the section was returned to the same stage coordinates, and imaged for both hypoxia (EF5) and total vasculature (antipanendothelial cell antigen, Pharmingen, San Diego, CA, USA). The total vasculature and perfused vasculature images were enhanced using colour segmentation to identify appropriate blood vessels (Fenton *et al*, 1999). Using ‘distance map’ filtering of the segmented images, individual pixel intensities were converted to levels directly proportional to the distances between tumour cells and the nearest blood vessel (Fenton *et al*, 2002). Percentage vascular area (defined as total or perfused vessel area/total tissue area) was also determined using Image Pro software. Finally, fluorescent image montages of the EF5/Cy3 staining were quantified by determining the mean pixel intensity of each image (range: 0–255). CCD camera settings were set to a constant shutter speed of 1/60, with constant gain, contrast, and brightness settings.

### Statistical analysis

Tumour means were compared using the Student's *t*-test and differences were considered significant for *P*<0.05.

## RESULTS

### Overexpression of VEGF_121_, VEGF_165_, or FGF-1 by MCF-7 cells enhanced oestrogen-dependent tumour growth

To determine whether expression of VEGF_121_, VEGF_165_, or FGF-1 by MCF-7 cells enhances MCF-7 breast tumour growth *in vivo*, MCF-7 vector controls, VEGF_121_, VEGF_165_, or FGF-1 cells were implanted orthotopically. At 45 days postimplantation, 40% of mice that received MCF-7/vector cells developed tumours, and volumes averaged 242±50.6 mm^3^. In contrast, expression of VEGF_121_, VEGF_165_, or FGF-1 not only increased the frequency of MCF-7 tumour formation but also dramatically enhanced tumour growth. As summarised in Table 1

**Table 1:** Tumour formation percentage and tumour volume at 45 days postimplantation

**Frequency (%)**	**Tumour formation**	**Tumour volume (mm^3^) Mean±s.e.**
Vector	40	240±50
VEGF_121_	90	1260±210
VEGF_165_	86	1640±190
FGF-1	74	760±120

, tumour volumes were significantly increased for each of the three transfectants in relation to vector controls (*P*<0.001).

### VEGF_121_, VEGF_165_, and FGF-1 have varying effects on tumour vascularity and perfusion

**Figure 1:**
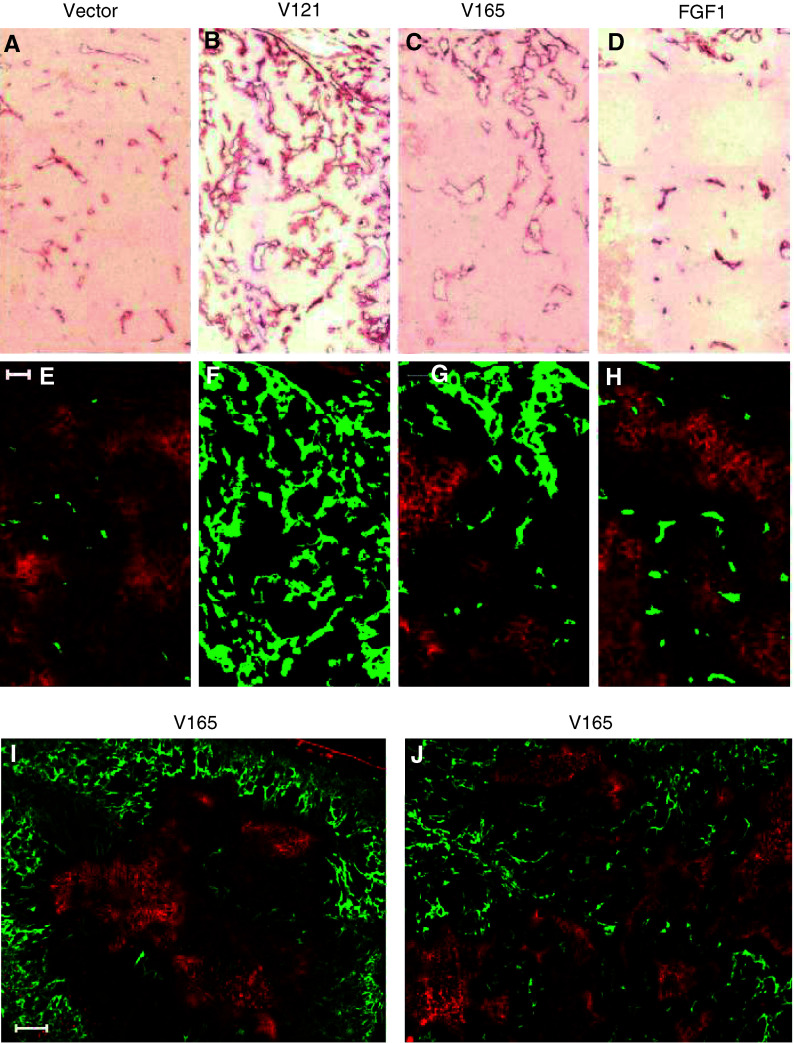
Representative immunohistochemical staining of antipanendothelial cell antigen in panels (**A**–**D**), with corresponding images of the DiOC_7_ perfusion marker (green) superimposed over the EF5 hypoxia marker (orange), in panels (**E**–**J**). Intensely stained orange regions of (**E**–**J**) correspond to increased tumour hypoxia. MCF-7 vector is shown in (**A**) and (**E**), VEGF_121_ in (**B**) and (**F**), VEGF_165_ in (**C**) and (**G**), and FGF-1 in (**D**) and (**H**). Each of panels (**A–H**) are portions of the original 4 × 4 composite images taken with a × 20 objective, and the bar in panel (**E**) equals 100 *μ*m. Panels (**I**) and (**J**) are entire 4 × 4 composites taken with a × 10 objective (bar in panel (**I**) equals 500 *μ*m), illustrating the two general patterns of vascular configuration and hypoxia observed in VEGF_165_ tumours. Peripheral vasculature with centralised hypoxia is shown in panel (**I**), and a more randomly distributed pattern of vasculature and hypoxia is shown in panel (**J**).

Since tumour vascularity and hypoxia have been shown to vary with tumour volume (Fenton *et al*, 1988), a separate set of volume-matched tumours was used for the pathophysiological measurements. The mean volumes±s.e. were as follows: vectors (410±90 mm^3^), VEGF_121_ (440±30), VEGF_165_ (570±80), and FGF-1 (440±90). Compared to vector controls, total vessel spacing was significantly decreased in both the VEGF_121_ (*P*<0.001) and VEGF_165_ (*P*=0.001) tumours, but unchanged in the FGF-1 tumours (see Figures 1A–D

and 2A

**Figure 2:**
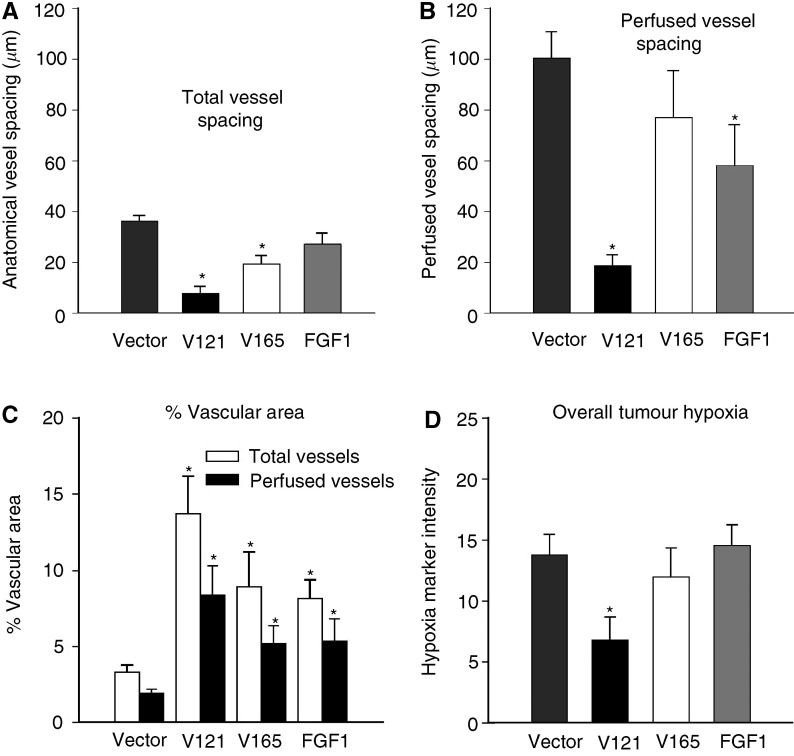
Effects of VEGF isoforms and FGF-1 on vascular spacing, % vascular area, and overall hypoxia. Data are presented as median distances (mean±s.e.) to the nearest total (**A**) or perfused (**B**) blood vessel, and increased median distances correspond to decreased vascular densities. An increased disparity between the total and perfused bars for a given tumour type indicates an increased proportion of nonfunctional vessels in that tumour. Data are averaged over four 4 × 4 image montages (64 fields) from each of 10 MCF-7 vectors (mean volume±s.e.m.=410±90 mm^3^), six VEGF_121_ tumours (440±30)), five VEGF_165_ tumours (570±80), and nine FGF-1 tumours (440±90). (**C**) Percentage vascular area for total (open bars) and perfused (filled bars) vessels. Asterisks denote statistically significant differences from vector controls. (**D**) Tumour hypoxia, as measured by overall EF5/Cy3 intensity (mean±s.e.), again averaged over four 4 × 4 image montages.

). Note that this decrease in vascular spacing corresponds to the increased vascularity shown in Figure 1. Perfused vessel spacing (Figure 2B), on the other hand, was significantly decreased for both the VEGF_121_ (*P*<0.001) and FGF-1 (*P*<0.013) tumours, but not for the VEGF_165_ (*P*=0.15) (see Figures 1E–H for representative images of the green perfusion stain superimposed on the orange hypoxia stain). To determine whether vascularity or perfusion varied with depth into the tumour, additional low-power image montages (× 10 objective) were also acquired to contrast vascular spacing in the centre (defined by a circular region of diameter 3000 *μ*m) *vs* the periphery of the tumour cross-section. On average, neither total nor perfused vessel spacing varied significantly with depth into the tumour for any of the four tumour models (data not shown). However, two distinctly different vascular patterns were observed among VEGF_165_ tumours. In roughly half of these tumours, a central region of hypoxia developed that was surrounded by a densely vascularised peripheral rim of vessels (Figure 1I), but in the others, vessels were fairly evenly distributed (Figure 1J).

Vessel diameters and interconnectivity (× 20 objective) were also markedly different among the transfectants, as shown in Figures 1A–D. In comparison to vector controls, percentage areas of both total (open bars in Figure 2C) and perfused (filled bars in Figure 2C) vessels were significantly increased for each of the three transfectants, again most strikingly for the VEGF_121_ tumours.

### VEGF_121_ overexpression reduces overall tumour hypoxia

Overall tumour hypoxia was characterised by measuring the mean intensity of the Cy3 conjugated antibody to the EF5 hypoxia marker. As summarised in Figure 2D (and shown by the orange staining in Figures 1E–H), overall tumour hypoxia was unchanged in the VEGF_165_ and FGF-1 tumous, but significantly reduced in the VEGF_121_ tumours (*P*=0.026) compared to vector controls. This decrease in hypoxia for the VEGF_121_ tumours is in agreement with the striking decrease in perfused vessel spacing observed for these tumours (Figure 2B), which corresponds to a decrease in the distance oxygen must diffuse to reach the tumour cells most distant from the vessels. In the case of the FGF-1 tumours, however, overall tumour hypoxia was unchanged despite a significant (although less pronounced than in the VEGF_121_ tumours) decrease in perfused vessel spacing.

## DISCUSSION

While VEGF is well accepted as an important modulator of tumour growth and vascular development, specific pathophysiological alterations associated with the different VEGF isoforms are less well understood. The current work reaffirms the notion that different VEGF isoforms lead to distinct differences in tumour vascular structure when compared at the same implantation site. In addition, we found that such vascular changes are, in some cases, directly associated with alterations in tumour oxygenation.

In previous studies, results have varied widely in terms of both tumour growth rate and vascular density when different tumour models and implantation sites were considered. Guo *et al* (2001) demonstrated that microenvironmental factors may be important, comparing VEGF_121_- and VEGF_165_-transfected glioma cell lines implanted either subcutaneously (s.c.) or intracranially (i.c.). VEGF_165_ transfectants grew much more rapidly than wild type at either location, with a corresponding increase in vascular density at both. Interestingly, VEGF_121_ transfectants exhibited enhanced vessel growth only when implanted orthotopically in the brain.

Using transfected fibrosarcoma cell lines, Grunstein *et al* (2000) proposed a model in which the different VEGF isoforms preferentially recruit blood vessels to either the tumour interior or periphery. It was suggested that these vascular patterns could possibly relate to the diffusibility of the VEGF_121_ *vs* the VEGF_165_. In this model, VEGF_120_-overexpressing tumours tended to more effectively recruit systemic vessels, but failed to develop adequate internal vascularisation (Grunstein *et al*, 2000), while VEGF_164_ tumours were capable of inducing both external and internal vascular expansion. In human melanoma transfectants, overall growth rate of the tumours correlated only with the amount of secretable VEGF, rather than on which specific VEGF isoform was overexpressed (Yu *et al*, 2002). Although VEGF_121_ tumours were more densely vascularised at the tumour periphery (with more central necrosis), VEGF_165_ tumours produced a much more densely vascularised plexus of blood vessels overall.

In the current study, human MCF-7 cells were implanted orthotopically in the mammary fat pad. Growth rates of VEGF_121_ and VEGF_165_ transfectants were significantly higher than vector controls and essentially equal to each other, while FGF-1 tumours grew at a somewhat less rapid rate. Both VEGF_121_ and VEGF_165_ produced densely arcading networks of blood vessels of increased vascular diameter. In contrast to both the fibrosarcomas and melanomas, however, spatial heterogeneities in vascular spacing were generally not observed. On average, neither total nor perfused vascular spacing varied as a function of distance from the tumour surface for any of the MCF-7 transfectants, although roughly half of the VEGF_165_ tumours demonstrated a reduction in vasculature in the tumour centre compared to periphery. Also, in contrast to previous reports in other models, MCF-7 VEGF_121_ transfectants were much more evenly vascularised than the VEGF_165_, as measured by the reduction in vascular spacing. Although the reasons for these disparate findings are unclear, spatially dependent vascular heterogeneities could possibly be related to either specific implantation site or differences in tumour volume.

A key advantage in our method of measuring vascular spacing, rather than the more commonly reported ‘vessels field^−1^’ or ‘positive pixels mm^−2^', is that vascular spacing is more closely related to the ability of the blood vessels to uniformly supply the tumour with oxygen and nutrients. Especially in tumours containing an uneven distribution of vessels, determinations of mean vascular density can be highly misleading in terms of tumour oxygen delivery. For example, a tumour with a highly localised cluster of dense vascularisation could have an overall vascular density equal to that of a tumour having a reduced but homogeneous distribution of vessels. Clearly, microregional efficiencies in the delivery of either oxygen or chemotherapeutic agents would be quite different between the two. Such differences are apparent when using our ‘distance map’ measurements of vascular spacing, which depend on vessel number, size, and spatial distribution. Although neither perfused vessel spacing nor tumour hypoxia was significantly altered in the VEGF_165_ tumours, VEGF_121_ tumours demonstrated significant changes in both. This decrease in perfused vessel spacing suggests that these vessels are more efficiently distributed in the VEGF_121_ tumours, which is supported by the significant decrease in overall tumour hypoxia observed in these tumours.

Finally, FGF-1 transfectants have also been reported to form large, vascularised tumours and to confer a more malignant phenotype upon MCF-7 cells, without oestrogen supplementation (Zhang *et al*, 1997). In the current studies, FGF-1 overexpression led to a substantial increase in tumour growth rate, with a significant decrease in the perfused vessel spacing. Conceivably, this increase in perfused vasculature could translate to an increased opportunity for these tumour cells to invade into the circulation and metastasise (Zhang *et al*, 1997).

A major unanswered question raised by this and previous studies is why VEGF_121_ and VEGF_165_ isoforms have such disparate effects on vascular structure and function among different tumour models. Although tumorigenicity and vascular growth were increased by both in all of the previously cited tumour models, specific alterations in vascular morphology were distinctly different. Interestingly, it has been reported that while VEGF_121_ is the predominant form expressed in human breast carcinomas (Relf *et al*, 1997) and melanomas (Yu *et al*, 2002), the VEGF_165_ variant is predominant in glioblastomas (Berkman *et al*, 1993). This is intriguing in view of the fact that the vascular modification associated with VEGF_121_ or VEGF_165_ transfectants of the three tumour types do not necessarily follow this same pattern. In breast tumours, the predominant variant, VEGF_121_, was also the more effective in inducing extensive tumour vascularisation when overexpressed in that model. In melanomas and gliomas, however, an entirely different relationship holds true, and in each case, the predominant isoform is the less important in terms of promoting vascular development (Guo *et al*, 2001; Yu *et al*, 2002).

Previous studies have speculated that differences in vascular configuration between VEGF_121_ and VEGF_165_ may be related to variations in heparin binding, isoform size, or diffusivity (Guo *et al*, 2001; Yu *et al*, 2002). It has also been hypothesised that variations in isoform expression may confer differential advantages on tumours as they expand in the different sites, each of which may possess different requirements for neovascularisation (Grunstein *et al*, 2000). Further detailed studies are needed to determine whether vascular response is primarily dictated by the immediate microenvironment of the tumour, including proximity to nearby pre-existing host vessels, or instead related to local balances among additional angiogenic growth factors and inhibitors.

## References

[bib1] Berkman RA, Merrill MJ, Reinhold WC (1993) Expression of the vascular permeability factor/vascular endothelial growth factor gene in central nervous system neoplasms. J Clin Invest 91: 153–159 8380810 10.1172/JCI116165 PMC330009

[bib2] Bicknell R (1997) Mechanistic insights into tumour angiogenesis. In Tumour Angiogenesis, Bicknell R, Lewis CE, Ferrara N (eds) pp 19–28. Oxford: Oxford University Press

[bib3] Bruns CJ, Shrader M, Harbison MT, Portera C, Solorzano CC, Jauch KW, Hicklin DJ, Radinsky R, Ellis LM (2002) Effect of the vascular endothelial growth factor receptor-2 antibody DC101 plus gemcitabine on growth, metastasis and angiogenesis of human pancreatic cancer growing orthotopically in nude mice. Int J Cancer 102: 101–108 12385004 10.1002/ijc.10681

[bib4] Fenton BM, Lord EM, Paoni SF (2001) Effects of radiation on tumor intravascular oxygenation, vascular configuration, hypoxic development, and survival. Radiat Res 155: 360–368 11175672 10.1667/0033-7587(2001)155[0360:eoroti]2.0.co;2

[bib5] Fenton BM, Paoni SF, Beauchamp BK, Ding I (2002) Zonal image analysis of tumour vascular perfusion, hypoxia, and necrosis. Br J Cancer 86: 1831–1836 12087474 10.1038/sj.bjc.6600343 PMC2375413

[bib6] Fenton BM, Paoni SF, Lee J, Koch CJ, Lord EM (1999) Quantification of tumor vascular development and hypoxia by immunohisto-chemical staining and HbO_2_ saturation measurements. Br J Cancer 79: 464–471 10027314 10.1038/sj.bjc.6690072 PMC2362405

[bib7] Fenton BM, Rofstad EK, Degner FL, Sutherland RM (1988) Cryospectrophotometric determination of tumor intravascular oxyhemoglobin saturations: dependence on vascular geometry and tumor growth. J Natl Cancer Inst 80: 1612–1619 3193479 10.1093/jnci/80.20.1612

[bib8] Ferrara N (1999) Vascular endothelial growth factor: molecular and biological aspects. Curr Top Microbiol Immunol 237: 1–30 9893343 10.1007/978-3-642-59953-8_1

[bib9] Gerber HP, Kowalski J, Sherman D, Eberhard DA, Ferrara N (2000) Complete inhibition of rhabdomyosarcoma xenograft growth and neovascularization requires blockade of both tumor and host vascular endothelial growth factor. Cancer Res 60: 6253–6258 11103779

[bib10] Grunstein J, Masbad JJ, Hickey R, Giordano F, Johnson RS (2000) Isoforms of vascular endothelial growth factor act in a coordinate fashion to recruit and expand tumor vasculature. Mol Cell Biol 20: 7282–7291 10982845 10.1128/mcb.20.19.7282-7291.2000 PMC86282

[bib11] Guo P, Fang Q, Tao HQ, Schafer CA, Fenton BM, Ding I, Hu B, Cheng SY (2003) Overexpression of vascular endothelial growth factor by MCF-7 breast cancer cells promotes estrogen-independent tumor growth *in vivo*. Cancer Res 63: 4684–4691 12907650

[bib12] Guo P, Xu L, Pan S, Brekken RA, Yang ST, Whitaker GB, Nagane M, Thorpe PE, Rosenbaum JS, Su HH, Cavenee WK, Cheng SY (2001) Vascular endothelial growth factor isoforms display distinct activities in promoting tumor angiogenesis at different anatomic sites. Cancer Res 61: 8569–8577 11731444

[bib13] Lord EM, Harwell L, Koch CJ (1993) Detection of hypoxic cells by monoclonal antibody recognizing 2-nitroimidazole adducts. Cancer Res 53: 5721–5726 8242628

[bib14] Relf M, Lejeune S, Scott PE, Fox S, Smith K, Leek R, Moghaddam A, Whitehouse R, Bicknell R, Harris AL (1997) Expression of the angiogenic factors vascular endothelial cell growth factor, acidic and basic fibroblast growth factor, tumor growth factor beta-1, platelet-derived endothelial cell growth factor, placenta growth factor, and pleiotrophin in human primary breast cancer and its relation to angiogenesis. Cancer Res 57: 963–969 9041202

[bib15] Shweiki D, Itin A, Soffer D, Keshet E (1992) Vascular endothelial growth factor induced by hypoxia may mediate hypoxia-initiated angiogenesis. Nature 359: 843–845 1279431 10.1038/359843a0

[bib16] Yu JL, Rak JW, Klement G, Kerbel RS (2002) Vascular endothelial growth factor isoform expression as a determinant of blood vessel patterning in human melanoma xenografts. Cancer Res 62: 1838–1846 11912163

[bib17] Zhang HT, Craft P, Scott PAE, Ziche M, Weich HA, Harris AL, Bicknell R (1995) Enhancement of tumor growth and vascular density by transfection of vascular endothelial cell growth factor into MCF-7 human breast carcinoma cells. J Natl Cancer Inst 87: 213–219 7535859 10.1093/jnci/87.3.213

[bib18] Zhang H-T, Scott PAE, Morbidelli L, Peak S, Moore J, Turley H, Harris AL, Ziche M, Bicknell R (2000) The 121 amino acid isoform of vascular endothelial growth factor is more strongly tumorigenic than other splice variants *in vivo*. Br J Cancer 83: 63–68 10883669 10.1054/bjoc.2000.1279 PMC2374542

[bib19] Zhang L, Kharbanda S, Chen D, Bullocks J, Miller DL, Ding IYF, Hanfelt J, McLeskey SW, Kern FG (1997) MCF-7 breast carcinoma cells overexpressing FGF-1 form vascularized, metastatic tumors in ovariectomized or tamoxifen-treated nude mice. Oncogene 15: 2093–2108 9366526 10.1038/sj.onc.1201386

